# Enterprise Strategic Management From the Perspective of Business Ecosystem Construction Based on Multimodal Emotion Recognition

**DOI:** 10.3389/fpsyg.2022.857891

**Published:** 2022-03-03

**Authors:** Wei Bi, Yongzhen Xie, Zheng Dong, Hongshen Li

**Affiliations:** ^1^School of Management, Shandong University, Jinan, China; ^2^School of Business Administration, Dongbei University of Finance and Economics, Dalian, China; ^3^School of Economics and Management, Shandong Youth University of Political Science, Jinan, China

**Keywords:** multimodal emotion recognition, deep learning, attention mechanism, business ecosystem, enterprise strategic management

## Abstract

Emotion recognition (ER) is an important part of building an intelligent human-computer interaction system and plays an important role in human-computer interaction. Often, people express their feelings through a variety of symbols, such as words and facial expressions. A business ecosystem is an economic community based on interacting organizations and individuals. Over time, they develop their capabilities and roles together and tend to develop themselves in the direction of one or more central enterprises. This paper aims to study a multimodal ER method based on attention mechanism. It analyzes the current emotional state of consumers and the development direction of enterprises through multi-modal ER of human emotions and analysis of market trends, so as to provide the most appropriate response or plan. This paper firstly describes the related methods of multimodal ER and deep learning in detail, and briefly outlines the meaning of enterprise strategy in the business ecosystem. Then, two datasets, CMU-MOSI and CMU-MOSEI, are selected to design the scheme for multimodal ER based on self-attention mechanism. Through the comparative analysis of the accuracy of single-modal and multi-modal ER, the self-attention mechanism is applied in the experiment. The experimental results show that the average recognition accuracy of happy under multimodal ER reaches 91.5%.

## Introduction

Ecosystems have been the hottest topic in theory and practice in the last decade. As an important branch of the ecosystem field, the business ecosystem with unique theoretical attributes has gradually attracted the attention of the people and actively carried out theoretical exploration. Strategic emerging industries are based on major technological breakthroughs and major development needs, and represent the direction of technological innovation as a direction of industrial development. In recent years, with the development of AI, the application of deep learning and the continuous update of computer science, more intelligent terminals have appeared in people’s field of vision, bringing people convenient and considerate services. In the process of human development and communication, an important part of it is emotional expression. Emotion recognition (ER) combines speech processing, psychology, standard recognition and video image processing, it can be applied to various fields such as education, transportation and medical care. And emotions can be expressed in different ways. Since single ER often has shortcomings such as insufficient information utilization and low recognition accuracy, more and more researchers have begun to pay attention to multimodal ER.

In this information age, ER has broad prospects for development. It can collect multiple emotional information of people in different backgrounds with the help of computers. Based on this, it can respond appropriately by analyzing people’s emotional states and guessing people’s inner emotions. In some specific occasions, it can show the effect of killing two birds with one stone. Multimodal ER is complemented by the pooling of information between different modalities, thereby improving the final recognition rate. In the business ecosystem, ER technology can initially control the corporate strategy, while improving the efficiency of the company, it also reduces the cost of human and material resources. At the same time, it can also provide reference for other business ecosystems and provide ideas for enterprises to choose value models in the new business environment.

The innovations of this paper are: (1) The fusion method of multimodal ER is analyzed. Among them, the fusion method based on feature layer can effectively utilize the information between different modes. However, the fusion method adopted by the directly cascaded feature layers is only to splicing the output emotion feature vectors of each modality. In this paper, the attention mechanism is effectively introduced into the process of feature layer fusion according to the research needs, which ensures the accuracy and rationality of the multimodal ER results. (2) A multimodal ER based on self-attention mechanism and neural network is proposed. It uses the self-attention mechanism to analyze the text data with the most abundant and accurate information for the three modal data of the user’s text, image and sound.

## Related Work

In recent years, with the continuous development of artificial intelligence technology, people hope that computers can serve people with emotion, and ER is one of the key technologies. Rao KP studied the proposed computer vision system that would automatically analyze videos of learners to identify students’ comprehension levels from their facial expressions and head movements. In the first stage, facial features are extracted to generate feature vectors using Accelerated Robust Features (SURF). In the second stage, head motion (nodding, shaking, or still) is detected based on nostril tracking. However, his proposed system cannot give feedback in real time (Rao et al., 2019). Wei proposed to use a multimodal strategy to extract emotional features from facial expression images. The basic idea is to combine low-level empirical features and high-level self-learning features into a multimodal feature. The two-dimensional coordinates of facial key points are extracted as low-level empirical features, and high-level self-learning features are extracted by convolutional neural network (CNN). However, there is a lot of redundant data in the implementation of this technology (Wei et al., 2020). Saha T thoroughly investigated the role of emotion in the automatic recognition of DA in task-independent dialog in a multimodal framework (especially audio and text). DL-based multi-task networks have been developed for DAC and ER, incorporating attention to facilitate the fusion of different modalities. An open-source, benchmarked ER multimodal dataset, IEMOCAP, has been manually annotated for its corresponding DA. However, the consumption cost of this network will be relatively large, and the practicability needs to be improved (Saha et al., 2020). Zhao S first briefly introduces widely used emotion representation models and emotion modalities, then he summarizes existing emotion annotation strategies and corresponding computational tasks, describing the main challenges in MER. Furthermore, he proposes some representative methods on representation learning for each emotion modality, feature fusion for different emotion modalities, MER classifier optimization, and MER domain adaptation. However, these methods are not based on reality and lack operability (Zhao et al., 2021). Wang X proposed a two-level attention mechanism with a two-stage multi-task learning (2Att-2Mt) framework for facial emotion estimation only on static images. First, features of corresponding regions (location-level features) are automatically extracted and enhanced by a one-level attention mechanism. A bidirectional recurrent neural network (Bi-RNN) with self-attention (two-level attention) is then used to adaptively make full use of the relational features (hierarchical features) of different layers. However, the shortcoming of this study is the lack of specific case analysis (Wang et al., 2018). The bimodal ER system proposed by Manisha S consists of three main parts, such as audio processing, video processing and data fusion, for detecting human emotions. The fusion of visual information and audio data obtained from two different sources improves the ER rate by providing complementary data. The proposed method aims to classify 7 basic emotions from input videos. He takes audio and image frames from video input to predict a person’s final emotion, using an audio-visual dataset that is particularly suitable for studying multimodal emotion expression and perception. But he did not propose specific measures (Manisha et al., 2021). Yoo G This study proposes a system that can identify human emotional states from biological signals, aiming to improve the interaction between humans and computers to achieve effective human-machine capable of intelligent interaction. The proposed method is able to identify six emotional states, such as joy, happiness, fear, anger, despair, and sadness, which are widely used for ER purposes. However, the accuracy of this experiment is not enough and the performance is low (Yoo et al., 2018). Kim J H proposed a multi-level fusion method to combine visual information and physiological signals for ER. For visual information, he proposes serial fusion of two-stage features to enhance the representation of facial expressions in video sequences. He proposes to combine neural aggregation networks with CNN feature maps to strengthen important emotional frameworks. For physiological signals, he proposed a parallel fusion scheme to widen the labeling range of EEG signals. However, the experiments in the article lack a lot of data (Kim and Lee, 2021).

## Multimodal Emotion Recognition and Deep Learning Related Methods

### Multimodal Feature Fusion Method

**FIGURE 1:**
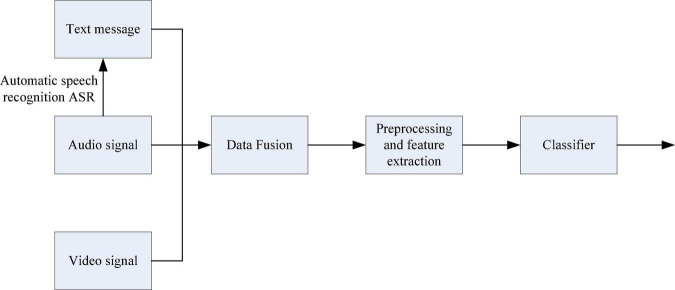
Data layer fusion flow chart.

**FIGURE 2:**
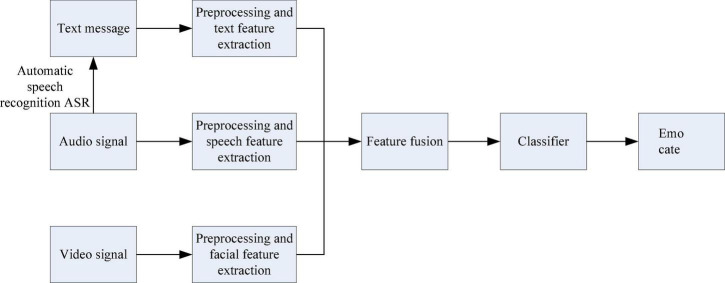
Feature layer fusion flow chart.

**FIGURE 3:**
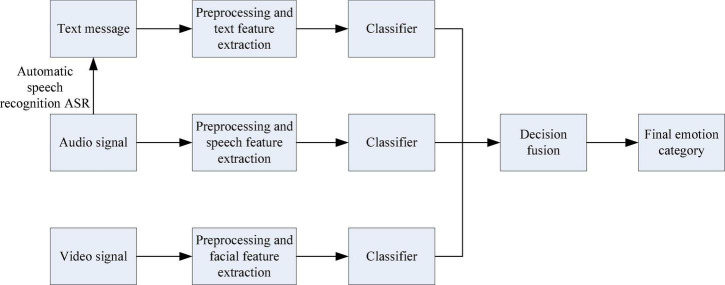
Flow chart of decision-making level fusion.

Although multimodal ER can well overcome the single shortcomings of single-modal ER, it is difficult to process and combine information in different ways. The traditional multimodal information fusion method framework includes data layer fusion, feature layer fusion and decision layer fusion, and the flow charts are shown in Figures 1–3, respectively (Min et al., 2018).

Data layer fusion refers to the direct fusion of the collected raw data layers, and the raw data of various emotional information is prepared in advance for data synthesis and analysis. Data layer fusion generally adopts a centralized fusion system for fusion processing.

Feature layer fusion is a fusion that belongs to the middle level. It first extracts effective features from the collected raw information, and then comprehensively analyzes and processes the feature information. The advantage of feature layer fusion is that considerable information compression can be achieved, which is beneficial to the real-time processing of the system. And because the extracted features are directly related to decision analysis, the fusion result can provide the feature information required for decision analysis.

Decision layer fusion is to perform preprocessing and feature processing on the single-modal information one by one, and then obtain the respective classification results through the classifier, and then fuse the respective classification results according to a certain form to obtain the final sentiment classification result. Common decision fusion criteria include: maximum, minimum, sum, average, voting, and product, etc. The fusion of the decision layer is simpler than the fusion of the data layer and the feature layer, because the dimensions of the classification results of each modal are consistent.

Comparing the three recognition frameworks for ER, they have their own advantages. However, practical problems must be considered in practical work in order to choose the best fusion method.

### Deep Learning Related Technologies

In multimodal ER, deep neural networks are widely used. Commonly used ones include: long short-term memory network (LSTM), CNN, and attention mechanism. In previous studies, these kinds of networks have achieved good recognition results. In this section, they will be briefly introduced.

(1) Long and short-term memory network.

**FIGURE 4:**
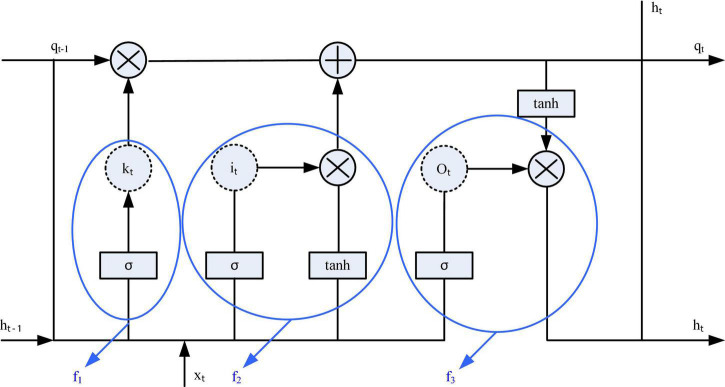
Long short-term memory network (LSTM) cell structure.

The Recurrent Neural Network (RNN) network model was first proposed in the 1980s, and now this network model has been widely used in various research fields (Zheng et al., 2019). Because the RNN can make good use of past information, it can be used as an important reference for current decision-making. Therefore, compared to other traditional models, this model can better utilize timing information that other models cannot. But at the same time, a new long-term dependency problem arises, which prevents the RNN from learning information that is relevant for a longer time (Rossi and Ruocco, 2019; Barabanschikov and Suvorova, 2020). In order to better solve the long-term dependence problem generated by RNNs, in 1997, some scholars proposed a new neural network structure–LSTM. The LSTM network is a special network structure with three “gate” structures. Figure 4 is a structural block diagram of the LSTM network.

The long-short-term memory network relies on the “gate” structure to effectively control the transmission information in the input network, thereby affecting the state of each moment in the neural network. Gate is a structure that combines a sigmoid neural network and a point multiplication function. There are three Gates in LSTM that work together, namely: forget gate, input gate and output gate. The key to solving the long-term dependency problem lies in the “forgetting gate” and the “input gate,” which allow the LSTM network to retain long-term memory.

In short, the forget gate can discard useless information, so that the neural network can forget the previous invalid information. The current input xt, the state qt-1 at the previous moment, and the output ht-1 at the previous moment will jointly decide which information needs to be forgotten by the forgetting gate. After discarding invalid relevant information, it is necessary to supplement the network with appropriate information. At this point, the forget gate comes into play. Similarly, according to xt, qt-1, ht-1 will jointly decide which information needs to be supplemented by the input gate into the current state qt. The function of the input gate is to make a valid output. According to the latest state qt updated by the network, the output ht-1 at the previous moment, and the current input xt, the output gate will generate the output ht at this moment. Through the “three-gate structure,” LSTM can effectively control the transmission state, discard unimportant information, and retain useful long-term memory. The specific work flow is described as follows:

In the LSTM network, the first step is to filter out the information that is not needed in the LSTM and discard it.


(1) $$f_{1}=\phi \,1\,\left(W_{1}\times x^{{}^{*}}\right)$$

Where $x^{{}^{*}}\,\overset{\Delta }{=}\left[x,s^{\left(o\,l\,d\right)}\right]$ means that the current input sample is connected to downstream time channel *s*^(*old*)^.

The second step is to supplement some new information in the LSTM. The sigmoid layer of the input gate f2 determines the information that needs to be updated, and the tanh layer is used to generate the updated alternative content.


(2) $$f_{2}=\phi \,1\,\left(W_{2}\times x^{{}^{*}}\right)•\varphi \,2\,\left(W_{3}\times x^{{}^{*}}\right)$$

The third step is to output information. The output gate f3 uses the sigmoid layer to determine the state of the output unit, which is processed first and then multiplied by the tanh layer, and finally output.


(3) $$f_{3}=s^{\left(n\,e\,w\right)}=\phi \,2\,\left(h^{\left(n\,e\,w\right)}\right)•\varphi \,1\,\left(W_{4}\times x^{{}^{*}}\right)$$

Among them, W1∼W4 represent the weight matrix corresponding to each gate.

The LSTM network relies on its unique three-gate structure to effectively solve the long-term dependency problem of the neural network. It is suitable for the modeling of speech time signals and text signals that are closely related to time.

### Convolutional Neural Network

**FIGURE 5:**
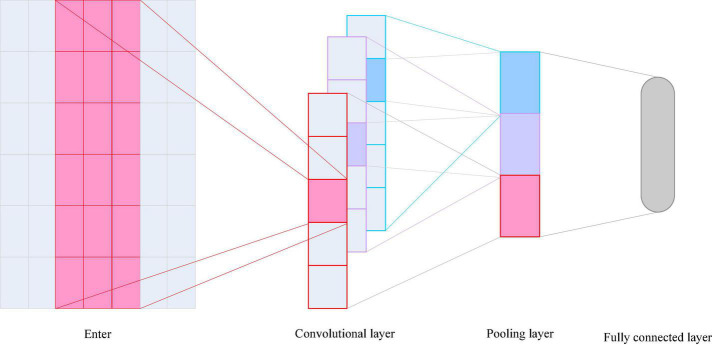
Convolutional neural network (CNN) basic network structure diagram.

Convolutional Neural Networks is one of the representative algorithms of deep learning. CNNs are widely used in speech-related tasks such as image classification, object detection, and speech synthesis. In the speech ER task, researchers also use CNNs to extract emotion-related speech features for ER. The basic structure includes convolutional layers, pooling layers, and fully connected layers, as shown in Figure 5.

Convolutional neural networks can automatically extract some advanced features, usually using backpropagation algorithm for parameter training and learning. The convolutional layer is the feature extraction unit of the CNN. The convolutional layer is composed of a convolutional kernel filter. The convolutional kernel parameters determine the convolutional layer to extract specific features. The parameters of a convolutional layer include: the number of input channels, the number of output channels, the size of the convolution kernel and the stride. The convolution kernel responds to local input features rather than the entire input feature map, and the local area where the response is obtained is the receptive field. In a feature map of a convolutional layer, the weights of the convolutional kernels are shared, which can detect specific patterns in different regions and greatly reduce the network parameters. The pooling layer often adopts mean pooling or max pooling to reduce noise and extract more robust features (Li et al., 2018).

The net input to the convolutional layer is calculated as follows:


(4) $$Z^{p}=W^{p}⊗X+b^{p}=\sum_{d=1}^{D}W^{p,d}⊗X^{d}+b^{p}$$

Where W represents the convolution kernel, X represents the feature map, and b represents the bias term.

After the network input is operated by a non-linear activation function, the feature output map *Y^p^* is obtained.


(5) $$Y^{p}=f\,\left(Z^{P}\right)$$

Where f(⋅) is the non-linear activation function.

Suppose the input of the convolutional layer is m, and the output of the l-th feature map of the convolutional layer is *n^l^*. The formula is expressed as:


(6) ni⁢jl=∑a=1A∑b=1B∑c=1Cωa⁢b⁢cl(ki+a)m(kj+b)+βi⁢jl

Among them, ω*^l^* represents the convolution kernel, and its size is A × B × C, A and B are the spatial dimensions, respectively, and C is the number of input channels. β*^l^* is the bias term, and k is the step size of the convolution kernel.

The expression for mean pooling is:


(7) $$z_{i\,j}=\frac{1}{A\,B}\,\sum_{a}\sum_{b}n\,\left(k\,i+a\right)\,\left(k\,i+b\right)$$

The expression for max pooling is:


(8) $$z_{i\,j}={\text{max}}_{a,b}\,n\,\left(k\,i+a\right)\,\left(k\,j+b\right)$$

Where A and B are the size of the pooling layer, and k is the stride of the pooling layer.

The activation layer provides non-linearity to the model. In general, the convolutional layer is followed by the activation layer. The normal activation functions include ReLU activation function, Sigmoid function and tanh function, etc. The formula expressions are as follows:


(9) R⁢(x)=max⁡(0,x)

(10) $$\sigma \,\left(x\right)=\frac{1}{1+e^{-x}}$$

(11) $$T\,\left(x\right)=\frac{e^{x}-e^{-x}}{e^{x}+e^{-x}}$$

Typical CNNs, such as AlexNet, VGGNet, and ResNet, etc., due to the existence of fully connected layers in their structure, the input must be a fixed-size image (No author Listed, 2017; Cai et al., 2019). The difference is that AlexNet uses a large-size convolution kernel design, and there is a grouping operation in the convolution layer. All convolutional layers of AlexNet are followed by ReLU-type activation functions, and the two convolutional layers starting after ReLU use local response regularization. The calculation of the local response normalization is shown in formula (12).


(12) $$b_{x,y}^{i}=a_{x,y}^{i}\,l\,{\left(k+\alpha \,\sum_{j=\max\left(0,i-n/2\right)}^{\min\left(N-1,i+n/2\right)}{\left(a_{x,y}^{j}\right)}^{2}\right)}_{\beta }$$

In formula (12), $a_{x,y}^{i}$ represents the activation value after passing through the ReLU function after computing the response with the convolution kernel i at position (x,y). $b_{x,y}^{i}$ is the output of local response regularization, and the total number of convolution kernels in this convolutional layer is N. The summation formula here is performed on the feature maps of n adjacent kernel functions at the same position, in order to suppress the output of those convolution kernels with larger output values. The constants k, n, α and β here are hyperparameters determined from the validation set. In AlexNet, *k* = 2, *n* = 5, α = 10e-4, β = 0.75.

(3) Attention mechanism.

The attention mechanism first appeared in the field of images, just as people pay more attention to the parts of things when they observe things. The principle of the attention mechanism is to assign high numerical weights to information units that have a great influence on the results, that is, to ensure that important information is not covered by irrelevant information through training parameters. Usually models that process sequence data, such as RNN and LSTM, use max pooling layers or average pooling to extract contextual information. Doing so often brings some losses, and using the max pooling layer may get misjudged information. The operation of average pooling often blurs the target and weakens the useful information.

Take the attention mechanism in machine translation as an example to illustrate in detail. The input sample sequence is sent to the encoder in time sequence, and the hidden state of the encoder is extracted at the last moment to initialize the hidden state of the decoder. The output sequences are then generated one after the other in time series. This encoder-decoder structure achieves better results than purely statistical based methods. But this RNN-based structure still has two serious flaws. First, RNNs are forgettable, which means that after thousands of temporal states, old information is forgotten. Second, there is no explicit word segmentation during decoding, so the system’s attention is spread over the entire input. To address these two issues, researchers introduced an attention mechanism into machine translation (Sarvestani and Boostani, 2017). They keep the RNN encoder, and during decoding, for each temporal state j, they compute an attention weight α_*ji*_ for each hidden state vector $h_{i}^{i\,n}$ of the encoder, and finally get a content vector *r*_*j*_:


(13) $$e_{j\,i}=a\,\left(h_{j}^{i\,n},h_{j}^{o\,u\,t}\right)$$

(14) $$\alpha_{i\,j}=\frac{e_{i\,j}}{\sum_{i}e_{i\,j}}$$

(15) $$r_{j}=\sum_{i}\alpha_{j\,i}\,h_{i}^{i\,n}$$

Here *r*_*j*_ is the weighted average of the input sequence elements, which is the encoded expression of the entire sentence for the current element $h_{j}^{o\,u\,t}$. $a\,\left(h_{i}^{i\,n},h_{j}^{o\,u\,t}\right)$ is the segmentation function, which is used to measure the similarity between two vectors. Then, *r*_*j*_ combines the current hidden state vector *h*_*j*_ and the output vector *y*_*j–1*_ of the previous step to generate the current moment output *y*_*i*_4:


(16) $$y_{i}=f_{y}\,\left(h_{j}^{o\,u\,t},y_{j-1},r_{j}\right)$$

(17) $$h_{j+1}^{o\,u\,t}=f_{h}\,\left(h_{j}^{o\,u\,t},y_{j}\right)$$

Among them, *f*_*y*_ and *f*_*h*_ represent the output layer and hidden layer functions of the RNN. This process is repeated until the end of the output sequence. By introducing this attention mechanism, the weak memory of RNN can be compensated. Because we compute the attention weight for each element in the input sequence, encoding vector *r*_*j*_ is not affected by the length of the input sequence. On the other hand, in this process, different attention weights are assigned according to the importance of different positions of the input sequence to the current decoding, and the soft localization of the input words can be achieved through this weight. This attention-based machine translation model has achieved great success.

The basic form of the attention mechanism is summarized as:


(18) $$e_{i}=a\,\left(u,\upsilon_{i}\right)$$

(19) $$\alpha_{i}=\frac{e_{i}}{\sum_{i}e_{i}}$$

(20) $$c=\sum_{i}\alpha_{i}\,\upsilon_{i}$$

Formula (18) calculates the attention weights, (19) normalizes the weights, and (20) encodes them according to the attention weights. First, u is a vector related to the task, which is matched according to the segmentation function *a*(*u*,υ_*i*_) and the i-th element υ_*i*_ in the sequence, and then the output weight scalar *e*_*i*_ indicates the degree of matching.

### Strategic Implications of Business Ecosystem Enterprises

American strategist first proposed the concept of “business ecosystem” (Liu et al., 2021). He defines a business ecosystem as an economic union based on the interaction of organisms and individuals. The definition was then further refined. It is considered to be a global network system, a real physical system, composed of business context and WWW-related abiotic factors, a virtual habitat system for this real system (Aung et al., 2017). Others emphasize the structure and interconnection of business ecosystems, that is, interconnected, dynamic businesses that organize consumers’ customers into business ecosystems.

## Multimodal Emotion Recognition Experiment and Analysis Based on Self-Attention Mechanism

### Experimental Dataset

Multimodal ER is an emerging field of research. Human communication and emotional expression are inherently multimodal. From a resource point of view, we really need large-scale datasets to deeply study multimodal ER (Alam et al., 2017). In this experiment, CMU-MOSI and CMU-MOSEI are the datasets we selected, and the data are summarized in Tables 1, 2.

**TABLE 1:** Statistical overview of the CMU-MOSI dataset.

Total number of video clip samples	2,199
Different speakers	89
Total number of videos	93
The average number of video clips cut by the video	23.2
Average length of video clip samples	4.2 s
The total number of words in the video clip sample	26,295
The total number of unique words in the video clip sample	3,107

**TABLE 2:** Statistical overview of the CMU-MOSEI dataset.

Total number of video clips	23,453
Total number of videos	3,228
Number of different speakers	1,000
Number of different topics	250
The number of video clips in a video	7.3
Average duration of each video clip	7.28 s
The total number of words in the video clip sample	447,143
The total number of unique words in the video clip sample	23,026

The first dataset is a video dataset MOSI7I (Multimodal Opinion-Level Sentiment Intensity) of 93 people commenting on certain topics in English, the videos are full of emotions. Each segment of the video was scored by 5 annotators based on the sentiment expressed by the segment, ranging from –3 for extremely negative sentiment to +3 for extremely positive sentiment. In this paper, the five annotation scores are averaged, and this average result is used as the sentiment polarity. That is, only two categories of positive emotions and negative emotions are considered. The videos of the first 62 people in the dataset are used as the training set, and the videos of the remaining 31 people are used as the test set. The training and test sets have 1,447 and 752 video clips, respectively.

### Recognition Design Under the Self-Attention Mechanism

To demonstrate the strengths of the models presented in this chapter, these two datasets are compared with classic models that have been used to solve multimodal sentiment analysis problems very well. There are three models that need to be compared: Simple LSTM (Simple Long Short-Term Memory Neural Network), which is proposed by the paper in ACL2017 and can represent a class of algorithm models that complete multimodal fusion through learning; Hierarchical RNN (RNN with hierarchical structure), the idea of this method is to splicing vectors of multiple modalities to complete multi-modal sentiment analysis; CAT-LSTM (Contextual Attention-Based LSTM), which is a multimodal fusion model based on attention mechanism (Sirai et al., 2017).

**FIGURE 6:**
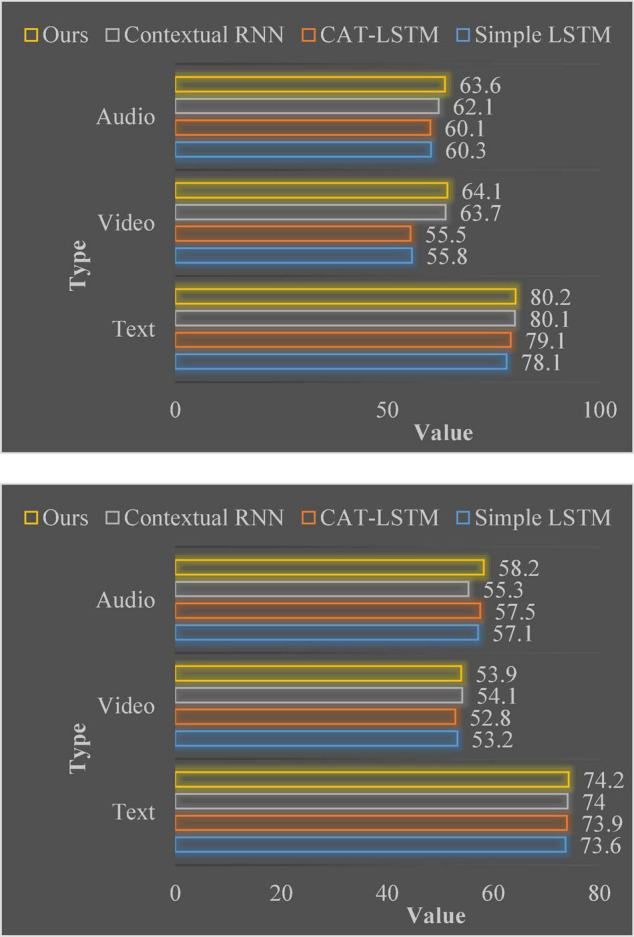
The accuracy of single-modal emotion analysis in MOSI and MOSEI, respectively.

Figure 6 shows the results of running all methods when only unimodal data is input. The self-attention-based unimodal model proposed in this chapter achieves better results than other basic models. This result suggests that self-attention mechanisms can be more effective than LSTM-based models, such as Simple LSTM. Because the self-attention mechanism can obtain more contextual information between video clips.

The self-attention mechanism can be applied to unimodal sentiment analysis. In order to further illustrate that this method can improve the accuracy of sentiment analysis by extracting contextual information between video clips in a single modality, this chapter conducts a single-modal sentiment analysis experiment to evaluate the model proposed in this chapter. Since the algorithm structure provided by Hierarchical RNN and Simple LSTM in single-modal sentiment analysis is exactly the same, the results of Hierarchical RNN are no longer written into the table. This chapter selects the Contextual RNN proposed in the paper published on EMNLP 2018 as another basic model for comparison. For this model, it is only necessary to apply the self-attention mechanism and Bi-GRU to three modalities of text, image and audio. The goal of the unimodal sentiment analysis experiment is to observe the effect of applying the self-attention mechanism to sentiment analysis.

**FIGURE 7:**
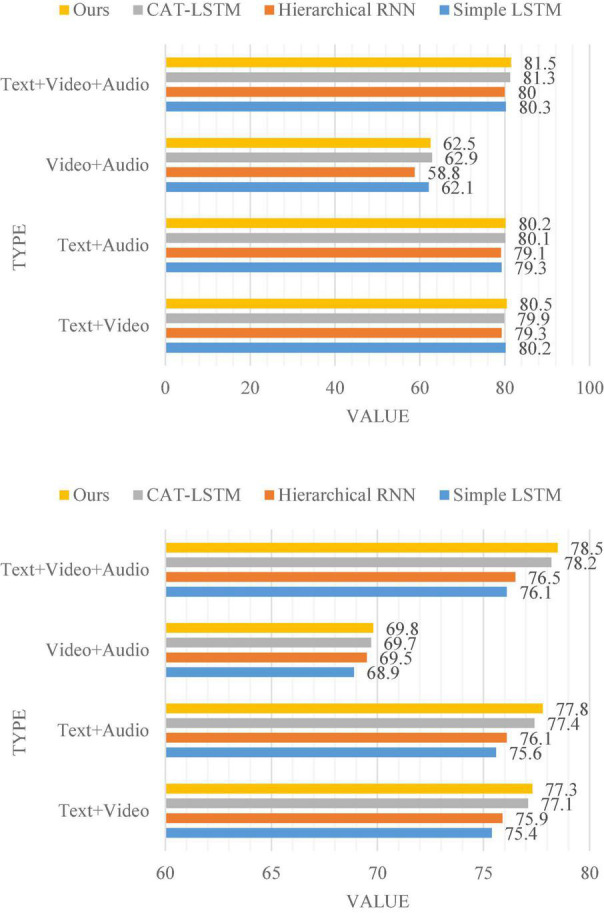
The accuracy of multimodal sentiment analysis in MOSI and MOSEI, respectively.

The results of the multimodal sentiment analysis comparison experiment are shown in Figure 7. We tested the sentiment analysis accuracy of all methods under three modalities and two modalities.

The three-modal sentiment analysis and two-modal sentiment analysis of the method proposed in this chapter outperform the three classical models in Figure 7 on both datasets. It shows that the model proposed in this chapter can more effectively utilize multimodal information in multimodal sentiment analysis problems. It is especially worth noting that the accuracy of this model is higher than that of Simple LSTM (Zhou et al., 2021). This shows that learning the connections between video clips with self-attention can improve the performance of the model in multimodal sentiment analysis. Noting that the accuracy of this model is higher than all methods except CAT-LSTM when only the fusion experiments are performed on image modalities and audio modalities. The main reason for this phenomenon is because the self-attention mechanism is only applied to text modalities, but not to images and audios. If the text modality is not considered at all, the variant of this model will adopt the idea of two-modal fusion based on self-attention, and the performance of the variant model will match or even exceed the performance of CAT-LSTM. In the presence of text modalities, this model needs to prioritize text. The vectors in the attention matrices generated by the three modalities are only concatenated with the vectors corresponding to the same video clip in the text modalities, and then used for sentiment classification.

### Multimodal Emotion Recognition Scheme

**FIGURE 8:**
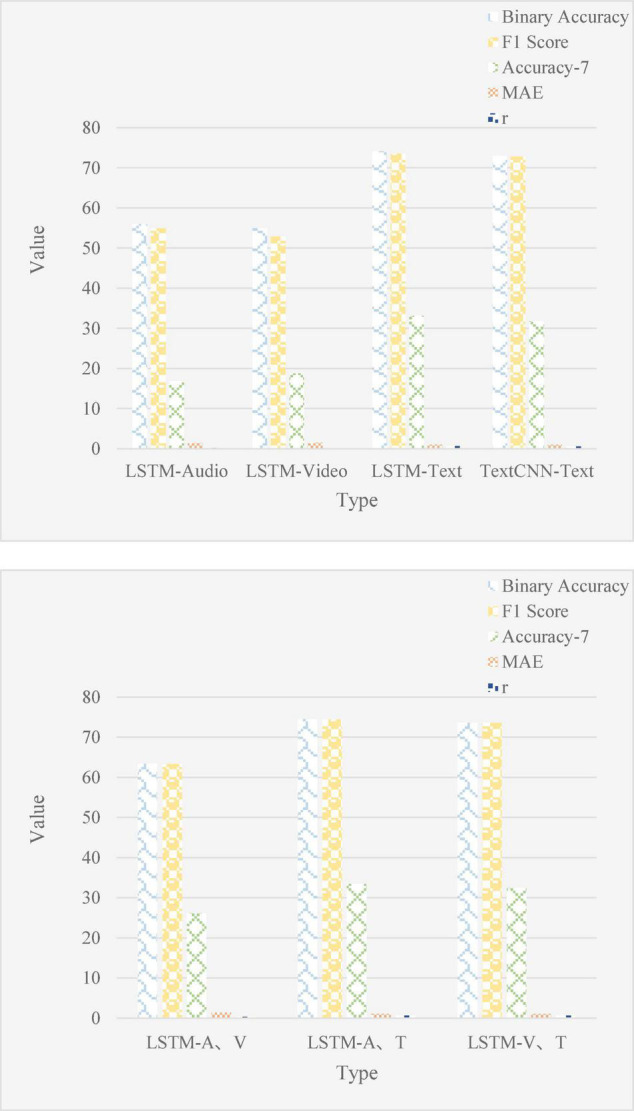
Single-modal and bi-modal emotion recognition (ER) results in MOSI dataset.

The single-modal and bi-modal ER results of the MOSI dataset are shown in Figure 8.

As can be seen from Figure 8, the accuracy of single-modal ER is generally low, especially in language and video. The accuracy of the binary classification is only 56.0 and 54.9%, while the text can reach 74.1%. In addition, for the text mode, the effect of using the Text CNN model for ER is not as good as that of LSTM+FC, and the Binary Accuracy is only 73.0%. From here, it can be analyzed that text as the main axis mode has a greater impact on ER, and compared to Text CNN, LSTM is also more suitable for our model. In the dual-modal ER results, LSTM-A and V are the models that use LSTM for cascade and FC processing using two modalities of speech and video. LSTM-A and T are two modes of speech and text, LSTM-V and T are two modes of video and text, and their models are the same as above. We will see that the BA of LSTM-A, V is 63.4%, the BA of LSTM-A, T is 74.5%, and the accuracy of LSTM-V, T is 73.6%. It can be clearly seen from the figure that the effect of dual-modal ER is higher than that of single-modal ER, and it can also be seen that the recognition results of related texts in dual-modal ER are relatively improved (Elleuch and Wali, 2019; Kim et al., 2021b; Liu and Fu, 2021).

The binary classification results report on the Confusion Matrix dataset for the test set on the MOSI dataset is shown in Table 3.

**TABLE 3:** MOSI test set Confusion Matrix.

Emotion category	Negtive	Positive	Total
Negtive	306	65	375
Positive	90	225	311
Total	396	290	686

**FIGURE 9:**
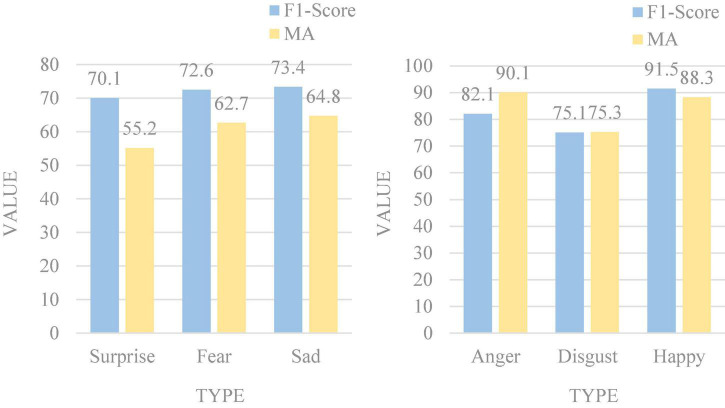
Emotion recognition (ER) results on the MOSEI dataset.

At the same time, we also performed 6-class multimodal ER on the MOSEI dataset, and Figure 9 is the recognition result.

It can be seen that the effect of multimodal ER is satisfactory. Among them, happy has the highest average recognition accuracy, reaching 91.5%, and surprise is the lowest, with an accuracy of only 55.2%. It can be seen that the recognition accuracy is generally more accurate than the original recognition, and the effect of multimodal ER is remarkable.

## Discussion

This paper is devoted to the study of multi-modal ER of deep learning, and three modalities of text, speech and video are selected. Firstly, a comprehensive analysis and overview of the background, significance, and related work of this paper are carried out, and the significance of this topic to enterprise strategic management from the perspective of business ecosystem is explained. Then, the multimodal feature fusion method and the important application of deep learning neural network in ER are introduced. Then, this paper uses deep learning neural network to identify single-modal and multi-modal emotional features. Finally, the multi-modal experimental results of this paper are described. This paper introduces the ER process that introduces the attention mechanism, and designs the analysis process of the experiment to compare the single-modal and multi-modal recognition results of this model (Khalaf et al., 2021; Kim et al., 2021a; Surendran et al., 2022).

In the business ecosystem, corporate strategic management faces a huge, non-centralized, borderless business environment, such as the network economy. When making strategic decisions for a company, it cannot just focus on a particular aspect of internal resources and closely related external companies. It cannot be guided solely by strategic management theory derived from this business environment. In the new era of entrepreneurial ecological environment, the practical orientation of traditional strategic management theory has been lost (Weisman et al., 2017). The perspective of enterprise management strategy should be “showing emotion from the scene,” and the focus of management strategy should be “like and love.” The strategic decision of an enterprise in a business ecosystem should be based on its value and connectivity characteristics, and according to which it forms the business ecosystem in which the enterprise is located. In this paper, a multimodal neural network model based on self-attention mechanism is established, and the review videos uploaded by users are taken as the research object, and sentiment analysis is performed on different parts of the videos. The experimental results are verified by comparing the experimental data of single-modal ER and multi-modal ER. On the basis of experiments, the analysis shows that the recognition of single modality is lower than the effect of multimodal ER, which proves that introducing additional methods will significantly improve the accuracy of ER.

## Conclusion

The vigorous development of science and technology has brought more development of artificial intelligence, and human-human interaction has turned to human-computer interaction. Therefore, people’s emotional requirements for artificial intelligence are getting higher and higher, and they want them to have the same emotions as people and can give more spiritual souls. Emotions are also a novel topic in both strategic and entrepreneurial studies. According to the concept and structure of the business ecosystem, the connection characteristics between the core enterprise and the complementary alternative enterprises in the business ecosystem will have an important impact on the strategic operation performance of the enterprise and the performance of the entire business ecosystem. The multimodal ER is based on the enterprise strategic management under the business ecosystem. It can make more accurate judgments on human emotions and better understand people’s psychological needs by reading human gestures, tone, facial expressions and other aspects of conveying emotions, so as to give correct and timely responses. In real life, there are many ways to express human emotions. When we talk, we make random expressions that match the words, the context, and often some body language. Therefore, the expressions of human emotions are diverse, and only a single modality will have a higher error rate, and the emotional judgment made by a single modality is far less than multimodal ER.

## Data Availability Statement

The original contributions presented in the study are included in the article/supplementary material, further inquiries can be directed to the corresponding author.

## Author Contributions

WB: writing. YX: editing. ZD and HL: data analysis. All authors contributed to the article and approved the submitted version.

## Conflict of Interest

The authors declare that the research was conducted in the absence of any commercial or financial relationships that could be construed as a potential conflict of interest.

## Publisher’s Note

All claims expressed in this article are solely those of the authors and do not necessarily represent those of their affiliated organizations, or those of the publisher, the editors and the reviewers. Any product that may be evaluated in this article, or claim that may be made by its manufacturer, is not guaranteed or endorsed by the publisher.
